# Pneumonia-targeted lopinavir/ritonavir-based treatment for patients with COVID-19: an early-period retrospective single center observational study

**DOI:** 10.1186/s12879-021-06588-5

**Published:** 2021-09-14

**Authors:** Jongkyu Kim, Jiwoong Jung, Tae Ho Kim, Naree Kang, Hanzo Choi, Dong Hyun Oh, Mi Young Ahn, Su hyun Kim, Chorom Hahm, Young Kyong Lee, Keunhong Park, Kiho Hong, Jae-phil Choi

**Affiliations:** 1 Department of Physical Medicine and Rehabilitation, Seoul Medical Center, Seoul, Republic of Korea; 2 Department of Surgery, Seoul Medical Center, Seoul, Republic of Korea; 3 Department of Internal Medicine, Seoul Medical Center, Seoul, Republic of Korea; 4 Department of Emergency Medicine, Kyunghee University at Gangdong, Seoul, Republic of Korea; 5 Department of Radiology, Seoul Medical Center, Seoul, Republic of Korea; 6 Department of Emergency Medicine, Seoul Medical Center, Seoul, Republic of Korea; 7 Department of Laboratory Medicine, Seoul Medical Center, Seoul, Republic of Korea

**Keywords:** Coronavirus disease 2019 (COVID-19), Severe acute respiratory syndrome coronavirus 2 SARS-CoV-2, Virus shedding, Lopinavir/ritonavir, Treatment outcome

## Abstract

**Background:**

Robust evidenced treatment strategy for Coronavirus disease 2019 (COVID-19) has not been established yet. Early, targeted, comprehensive management approach can be essential.

**Methods:**

A lopinavir/ritonavir (LPV/r)-based antiviral treatment was administered to the patients with computed tomography (CT)-documented pneumonia. Medical records of patients with COVID-19, previously discharged or hospitalized for ≥ 21 days at the Seoul Medical Center from January 29 to April 15, 2020 were reviewed to analyze clinical and virological outcomes. Patients were divided into two groups (PCR-Negative conversion group vs. Non-negative conversion group and requiring oxygen group vs. Non-requiring oxygen group).

**Results:**

In total, 136 patients with a mean age of 41.8 ± 18.2 years were included with median 3-day delay of hospitalization after illness. Thirteen (9.56%) were initially asymptomatic, and 5 (3.67%) were persistently asymptomatic. Eighty-five (62.5%) had CT-documented pneumonia, 94% of whom received LPV/r treatments. A total of 53 patients (38.97%) had negative polymerase chain reaction (PCR) results within 28 days. Eight (9.4%) out of 85 pneumonic patients received oxygen supplementation. Patients with initial lower respiratory symptoms showed significant delay in PCR negative conversion (> 28 days) (odds ratio [OR] 0.166; 95% confidence interval [CI] 0.067–0.477; *P* < 0.001). However, antiviral treatment for pneumonic patients was significantly related with early conversion within 28 days (OR 3.049; 95% CI 1.128–8.243; *P* = 0.028). Increasing age increased the likelihood of oxygen supplementation requirement in the pneumonic patient group (OR 1.108; 95% CI 1.021–1.202; *P* = 0.014).

**Conclusions:**

Early, pneumonia targeted LPV/r-based antiviral therapy resulted in a significantly higher probability of negative conversion of PCR within 28 days compared to symptomatic treatment.

## Background

The coronavirus disease 2019 (COVID-19) pandemic has overwhelmed the healthcare systems of many countries, with many cities reaching the near peak of their capacity in terms of the COVID-19 outbreak. Slowing down and flattening the peak are essential to ensure that current healthcare systems are able to cope with the sudden rise in patient volumes [1]. Reported clinical outcomes in cities outside Wuhan have varied; with data from Beijing indicating that 17.6% of patients were categorized as having severe pneumonia, and showing a 0.9% reported fatality rate [2, 3] and, 8.8% of patients were transferred to intensive care units in Shanghai [4]. Another study used data from three Chinese cities to compare patients of moderate severity (74%) with severe or critical patients [5].

Early case series from the Korean Cohort Study showed that 21.4% of patients received oxygen therapy, with no requirement for mechanical ventilation [6]. In Daegu and the Gyeongbuk area, Republic of Korea, substantial community outbreaks were linked to a large religious meeting. This crisis temporarily exceeded the capacity of the previously well-functioning healthcare system and resulted in a case fatality of 94% all national death [7]. Hospitals in the metropolitan Seoul also had to provide medical services to increasing number of patients with COVID-19 including 97 cases from a call center outbreak, many small sized cluster outbreaks, imported cases, and their contacts [8].

Here, we present the early period clinical outcomes of a pneumonia targeted lopinavir/ritonavir treatment for patients with COVID-19 in one of largest COVID-19-dedicated hospital in Seoul. We investigated the risk factors associated with prolonged viral detection or disease progression requiring oxygen supplementation.

## Methods

### Hospital reorganization in response to the COVID-19 outbreak

Seoul Medical Center is a 623-bed municipal general hospital located in northeastern Seoul. Ten national designated high-level negative pressurized isolation unit (HLIU) beds were activated once treatment began for the fifth patient with COVID-19. As nationwide outbreaks increased, to counteract the medical surge in metropolitan Seoul, all in-patient beds were mobilized, and all previously hospitalized patients were evacuated to other affiliated hospitals. To block air recirculation and to supply 100% of the outer air into a ventilation system, 109 isolation rooms were equipped with portable negative pressure machines. Through elevator control, a one-way entrance and exit system was designed, video monitoring systems operated in each room, and an intensified electronic medical chart monitoring system was established, which included a modified early warning score (MEWS), oxygen saturation, and fever monitoring [9]. Multi-disciplinary specialist doctors working as attending physicians, and nursing staff volunteered to treat hospitalized patients with COVID-19. Ten previously activated HLIUs were used as intensive care units (ICUs).

### Intervention and patient care protocol

#### Initial assessment and triage at the emergency department (ED)

At the ED, chest X-ray, chest low-dose computed tomography (LDCT) scans, and laboratory assessments were undertaken for all patients diagnosed with COVID-19 due to severe acute respiratory syndrome coronavirus 2 (SARS-CoV-2) infection. The patients were then categorized for admission to the ICU or a general ward using MEWS.

#### An in-hospital electronic medical record monitoring system

An in-hospital electronic medical record monitoring system (recording daily MEWS, oxygen saturation and fever monitoring, scheduled plain chest radiograph imaging, and alerts by one radiologist; YKL, with 20 years of clinical experience of thoracic imaging) was used to monitor patients showing signs of acute deterioration.

#### Protocol-based antiviral treatment

Antiviral agents were prescribed for patients with pneumonia, which was documented on an initial chest computed tomography (CT) scan, to reduce the severity of the disease or clinical deterioration. Lopinavir/ritonavir (LPV/r) 400 mg/100 mg twice daily was administered as a first line regimen [10]. If this regimen was contraindicated or not tolerated, it was replaced with hydroxychloroquine 400 mg once a day. The treatment duration was determined according to the clinician’s decision either for 7 or 10–14 days. A 10-day course of remdesivir clinical trial was also performed for consenting patients with radiologically confirmed pneumonia [11, 12]. Ciclesonide, two puffs, twice daily inhalation was used as an adjuvant treatment, as indicated by the attending physician [13].

### Study design and patients

Out of the 619 confirmed COVID-19 cases in Seoul, 251 patients with mild-moderate to severe COVID-19 were treated at the Seoul Medical Center by April 15, 2020.

In this study we retrospectively analyzed acute in-hospital clinical outcomes in a single-center from January 29, 2020 to April 15, 2020. The study comprised of data of 140 SARS-CoV-2-infected polymerase chain reaction (PCR)-confirmed patients, who had been discharged from the Seoul Medical Center or had been hospitalized for ≥ 21 days.

Investigators reviewed data in relation to all epidemiological and demographic patient characteristics, underlying co-morbidities, days from initial symptoms to admission, initial symptom manifestations or signs, laboratory data and radiological findings. Symptoms were categorized as asymptomatic, general (fever, chill, and myalgia), upper respiratory (sore throat, rhinorrhea, and nasal congestion), lower respiratory (cough, sputum, dyspnea, and chest discomfort), or others. The use of antiviral agents, PCR-negative conversion as a virological outcome, and clinical outcomes including the need for nasal oxygen supply, high-flow nasal cannula (HFNC), ventilator support, in-hospital days and case fatalities were assessed.

All the patients were diagnosed and serially followed using real-time reverse transcription PCR assays for the SARS-CoV-2 E gene and RNA-dependent RNA polymerase gene from sputum and nasopharyngeal specimens, using a PowerChek 2019-nCoV Real-time PCR Kit (Kogene Biotech, Seoul, South Korea). Negative conversion of both sputum and nasopharyngeal specimens for two consecutive days was considered as viral clearance. Observed PCR-negative conversion duration was defined as the duration from the symptom onset to the PCR-negative conversion for discharged patients. Patients hospitalized for > 21 days or transferred to other facilities without PCR-negative conversion were regarded as censored cases and the last hospital day was used to calculate observed duration.

The presence of any ground-glass opacity (GGO) or consolidation in the bilateral peripheral posterior lungs on chest CT scans without any other specified causes or pathogen in a COVID-19 confirmed patient was categorized as CT-documented COVID-19 pneumonia [14, 15]. Clinical deterioration or disease progression was defined as following conditions after treatment for ≥ 3 days; (1) persistent or newly developed fever, (2) radiological aggravation of a previous lesion or newly developed opacity and (3) requiring oxygen therapy or vasopressor.

### Statistical analysis

Continuous variables were represented as mean and standard deviation (SD) or median and inter-quartile ranges (IQR). The one-sample Kolmogorov–Smirnov test was performed to check the normality of all variables. The patients were divided twice into two groups (first, PCR-Negative conversion group vs. Non-negative conversion group and then requiring oxygen group vs. Non-requiring oxygen group). To compare the two groups, Mann–Whitney U test was used for continuous variables and chi-square or Fisher’s exact test was used for categorical variables. Statistically significant variables were chosen from the comparison of the two groups and multiple logistic regression analyses were performed to identify the clinical variables associated with negative conversion and oxygen requirement.

All statistical analyses were performed using the SPSS statistical software version 25.0 (IBM Corp., Armonk, NY, USA). P values were based on a two-sided significance level of 0.05.

## Results

### Basic characteristics of the enrolled patients

**Fig. 1:**
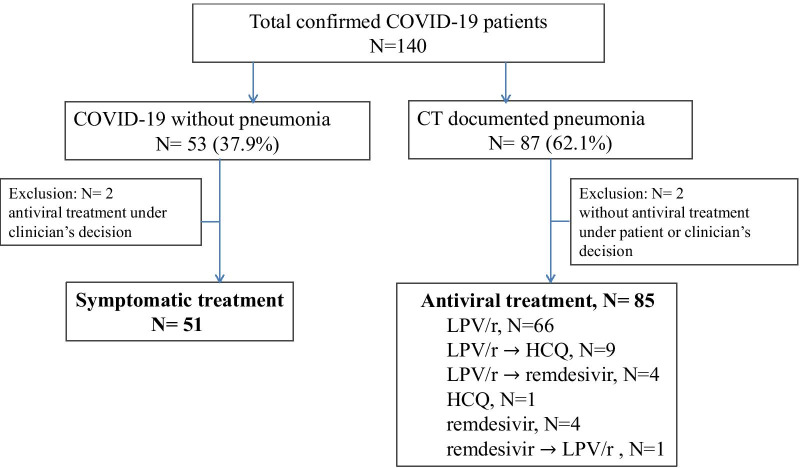
Treatment overview concerning 136 enrolled patients with COVID-19

From January 29, 2020 to April 15, 2020, 136 out of 140 PCR-confirmed patients with COVID-19, who had been discharged or had been hospitalized for > 21 days were included in this analysis (Fig. 1). The median time between symptom onset and the first antiviral treatment was 3 days. LPV/r was administered to 80 (94%) patients and, 86% of them completed the regimen without serious adverse events or clinical progression. Eight (10%) of 80 patients were switched to hydroxychloroquine due to adverse events (diarrhea or nausea, elevation of alanine aminotransferase, amylase, or creatinine kinase). In four (5%) patients, LPV/r was switched to remdesivir due to clinical deterioration. One patient on remdesivir discontinued treatment because of bradycardia and changed to LPV/r.

The mean patient age was (41.8 ± 18.2) years, 48.5% of whom were male. Thirteen (9.56%) patients reported no symptoms until the day of admission, and 3 of these were found to have pneumonia on an initial chest CT scan. A total of 5 (3.97%) out of 13 asymptomatic patients persistently remained asymptomatic during the entire in-hospital stay and the remaining 8 patients complained of newly developed cough, fever, headache, or sore throat within 2 days after admission.

The median observed duration from symptom onset to PCR-negative conversion was 29 days. At the time of analysis, 56% of the patients had been discharged and released from quarantine.

### Comparison between the PCR-negative conversion group and non-negative conversion group within 28 days

The PCR-negative conversion group included more patients with neurological disorder (15.1% vs 3.6%) than the non-negative conversion group (Table 1). Lower respiratory symptoms were less (11.3% vs 43.4%) and CT scan documented pneumonias were more common (77.4% vs 53.0%) in negative conversion group than the others. Otherwise including admission delay or initial laboratory findings were comparable (Table 2).

**Table 1:** Comparison of basic characteristics between the polymerase chain reaction-negative conversion group and non-negative conversion group within 28 days (*N* = 136)

Variables, number (%)	Negative conversion (*N* = 53)	Non-negative conversion (*N* = 83)	*P* value
Age, year (mean ± standard deviation)	43.7 ± 17.4	40.5 ± 18.7	0.324
Male gender	28 (52.8)	38 (45.8)	0.423
Epidemiological linkage			0.094
Imported cases	8 (15.1)	21 (25.3)	
Household contact	10 (18.9)	22 (26.5)	
Non-familial contact	15 (28.3)	20 (24.1)	
Cluster outbreak	12 (22.6)	17 (20.5)	
Unknown	8 (15.1)	3 (3.6)	
Co-morbidities			
Cardio-pulmonary diseases	9 (17.0)	21 (25.3)	0.254
Endocrine diseases	5 (9.4)	10 (12.0)	0.635
Neurological diseases	8 (15.1)	3 (3.6)	0.023
Other diseases	9 (17.0)	15 (18.1)	0.871
Delayed admission ≥ 3 days	33 (62.3)	49 (59.0)	0.708
Initial symptom			
Asymptomatic	4 (7.5)	9 (10.8)	0.524
General (fever, chill, and myalgia)	33 (62.3)	53 (63.9)	0.851
Upper respiratory (sore throat, rhinorrhea, and nasal congestion)	15 (28.3)	23 (27.7)	
Lower respiratory (cough, sputum, dyspnea, and chest discomfort)	6 (11.3)	36 (43.4)	< 0.001
Others	5 (9.4)	4 (4.8)	0.311
Pneumonia on initial chest computed tomography	41 (77.4)	44 (53.0)	0.004

**Table 2:** Initial laboratory findings between the polymerase chain reaction-negative conversion group and non-negative conversion group within 28 days (*N* = 136)

Variables, median (IQR)	Negative conversion (*N* = 53)	Non-negative conversion (*N* = 83)	*P* value
White blood cells, 10^9^/L	5.60 (4.20–7.35)	5.20 (4.45–6.50)	0.556
Lymphocyte, %	30.80 (24.25–36.90)	27.30 (19.60–34.10)	0.160
Neutrophil to lymphocyte ratio	1.87 (1.41–2.76)	2.17 (1.53–3.25)	0.258
C-reactive protein, mg/dL	0.33 (0.11–1.56)	0.17 (0.09–0.60)	0.180
Procalcitonin, mg/dL	0.04 (0.02–0.05)	0.04 (0.03–0.05)	0.244
Lactate dehydrogenase, U/L	244 (214–313)	240 (206–283)	0.246
Ferritin, μg/L	205.91 (120.61–441.07)	145.84 (71.04–284.62)	0.118

### Comparison of characteristics between pneumonic patients requiring oxygenation and those not requiring oxygenation

Among 85 pneumonic patients out of 136 patients, 8 (9.4%) required oxygen supplementation (Table 3). Of these, 4 patients were treated with HFNC and 2 patients required mechanical ventilation. In a univariate analysis, the group requiring oxygen consisted of older patients (mean ± SD, 73.6 ± 16.1 vs 45.9 ± 15.1 year) and had increased presentation of either cardio-pulmonary (62.5% vs 26.0%) or neurological diseases (50% vs 7.8%). The median initial C-reactive protein (6.67 vs 0.39 mg/dL), procalcitonin (0.28 vs 0.04 mg/dL), Lactate dehydrogenase (420 vs 254 U/L) and ferritin (634.86 vs 173.67 μg/L) were higher in the oxygenation requiring group compared to the non-oxygen requiring group.

**Table 3:** Comparison of basic characteristics between pneumonic patients requiring oxygenation and non-requiring oxygenation (*N* = 85)

Variables, number (%)	Requiring oxygen (*N* = 8)	Non-requiring oxygen (*N* = 77)	*P* value
Age, year (mean ± standard deviation)	73.6 ± 16.1	45.9 ± 15.1	< 0.001
Male gender	4 (50.0)	40 (51.9)	0.999
Co-morbidities			
Cardio-pulmonary diseases	5 (62.5)	20 (26.0)	0.045
Endocrine diseases	3 (37.5)	10 (13.0)	0.100
Neurological diseases	4 (50.0)	6 (7.8)	0.006
Other diseases	4 (50.0)	16 (20.8)	0.084
Delayed admission ≥ 3 days	4 (50.0)	55 (71.4)	0.241
Initial symptom			
Asymptomatic	0 (0)	3 (3.9)	0.999
General (fever, chill, and myalgia)	7 (87.5)	55 (71.4)	0.439
Upper respiratory (sore throat, rhinorrhea, and nasal congestion)	0 (0)	22 (28.6)	0.105
Lower respiratory (cough, sputum, dyspnea, and chest discomfort)	1 (12.5)	21 (27.3)	0.674
Others	1 (12.5)	6 (7.8)	0.513
Initial laboratory findings median (IQR)			
White blood cells, 10^9^/L	6.6 (4.42–13.8)	5 (4.15–6.45)	0.268
Lymphocyte, %	22.65 (7–33.52)	29.2 (22–35.5)	0.155
Neutrophil to lymphocyte ratio	3.04 (1.61–13.09)	2.05(1.46–2.97)	0.157
C-reactive protein, mg/dL	6.67 (2.04–9.47)	0.39 (0.14–1.12)	0.001
Procalcitonin, mg/dL	0.28 (0.04–0.68)	0.04 (0.03–0.05)	0.004
Lactate dehydrogenase, U/L	420 (317.25–532)	254 (215.5–309.5)	0.001
Ferritin, μg/L	634.86 (300.08–2482.79)	173.67 (85.13–364.48)	0.002

### Odds ratio associated with PCR negative conversion within 28 days or requiring oxygenation

A multivariate analysis revealed that patients who had presented with initial lower respiratory symptoms showed significantly lower odds ratio (OR) of virological negative conversion within 28 days (OR 0.166; 95% CI 0.061–0.454; *P* < 0.001) (Table 4). However, antiviral treatment for pneumonia (OR 3.049; 95% CI 1.128–8.243; *P* = 0.028) revealed increased likelihood of viral clearance.

**Table 4:** Final Adjusted Logistic Model of negative conversion within 28 days

Variable	Odds ratio	95% Confidence interval	*P* value
Age (per 1 year increase)	0.985	(0.960–1.011)	0.257
Male gender	1.208	(0.558–2.613)	0.632
Delayed admission ≥ 3 days (yes vs. no)	1.198	(0.527–2.726)	0.666
Lower respiratory symptom (yes vs. no)	0.166	(0.061–0.454)	< 0.001
Neurological diseases (yes vs. no)	4.818	(0.953–24.352)	0.057
Antiviral agent (use vs. non-use)	3.049	(1.128–8.243)	0.028

Final adjusted logistic model of the pneumonic patients requiring oxygenation showed that the likelihood of oxygen supplementation increased by 1 year (OR 1.108, 95% CI 1.021–1.202; *P* = 0.014) (Table 5).

**Table 5:** Final Adjusted Logistic Model of the pneumonic patients requiring oxygenation

Variables	Odds ratio	95% Confidence interval	*P* value
Age (1 year increasing)	1.108	(1.021–1.202)	0.014
Cardio-pulmonary diseases (yes vs. no)	0.964	(0.105–8.867)	0.974
Neurological diseases (yes vs. no)	3.421	(0.451–25.935)	0.234

## Discussion

Robust, evidenced treatment strategy for Coronavirus disease 2019 (COVID-19) has not been established yet. In this study, we presented the early period treatment outcomes of patients in one COVID-19 dedicated hospital involving a wide range of citizens in the Seoul metropolitan area. Rapid case detection and immediate hospital admission, CT scans to identify the patients at a risk of pneumonia, intensive monitoring, and early pneumonia targeted LPV/r-based antiviral treatment constituted the treatment strategy for patients with COVID-19.

A total of 90.4% of the patients were in the symptomatic group; among them 65.6% had CT documented pneumonia and 7.0% received oxygen treatment. In otherwise initially asymptomatic patients, 5 (3.97% of total) patients remained persistently asymptomatic during their entire hospital stay, and these patients would not have been detected using the current symptom-based surveillance system.

LPV/r, chymotrypsin-like protease inhibitor-based antivirals, were administered to patients with CT-documented COVID-19 pneumonia in our cohort. LPV/r was well tolerated in 81.3% of patients without serious adverse events or clinical deterioration, which required alteration to their medication. LPV/r-based randomized controlled study for severe COVID-19 pneumonia failed to show a reduction in the clinical improvement time [16]. In LPV/r arm, a numerically lower but not statistically significant 28-day mortality rate was observed with 13% of discontinuation. In our study, we did not use clinical improvement as an outcome, so it is hard to compare both studies. We agree with a claim that a median 13-day delayed randomization from illness onset to subsequent antiviral initiation is too late in terms of virus-host interaction and that considerable tissue damage could occur in patients (median lactate dehydrogenase level of 325 U/L; interquartile range, 245–433 U/L) [16, 17]. However, the median delay from the initial symptom to admission and subsequent antiviral treatment was 3 days and the median lactate dehydrogenase level was 243 (interquartile range 209–297) U/L in our study.

An early report from Wuhan by Zhou et al., which included non-survivors, showed that the median duration of SARS-CoV-2 shedding was 20 days [18]. Data from 113 symptomatic patients in a study by Xu et al. indicated that the median duration was 17 days [19]. The risk factors of prolonged virus shedding, which was defined as ≥ 15 days, included male sex, old age, hypertension, admission delay, severe illness at admission, invasive mechanical ventilation, and steroid treatment. However, Zhou et al.’s more recently published data showed that the median duration of viral shedding in 41 discharged patients was 31.0 days from illness onset [20]. The median observed duration of PCR-negative conversion in our study was 29 days, which was 12 days longer than that reported by Xu et al., but which was consistent with Zhou et al.’s findings. We arbitrarily used the median observed duration, which comprised of viral clearance days and the last hospital day for patients hospitalized for > 21 days.

We noticed that patients who initially presented with lower respiratory symptoms were significantly associated with a prolonged PCR-positive duration, whereas antiviral treatment in the presence of pneumonia resulted in a significantly higher probability of negative conversion within 28 days than symptomatic treatment only. Although longer viral shedding itself is not related with clinically severity, a longer duration of virus detection negatively affects public health, medical resource use, and the patient’s socioeconomic well-being. Some randomized controlled trials are currently underway, in which antiviral agents are being administered to patients in a mild COVID-19 group, for which our data are likely to provide support.

Increasing age was the significant risk factor for oxygen requirement. Cardiopulmonary or neurological comorbid diseases did not associate with the progression of the disease.

This study has some limitations. Firstly, this was a retrospective study to describe clinical experiences at the early stage COVID-19 outbreak in Seoul area; hence, the clinical efficacy of the pneumonia targeted LPV/r treatment cannot be elucidated. Secondly, some unrecognized confounders might have affected our results because our study was based on a relatively small population compared with the entire number of patients with COVID-19 in Korea, and our patient outcome findings were derived from a single center. However, the age distribution of the patients with COVID-19 in our cohort was representative of patients residing in Seoul. Thirdly, our population included a relatively small number of serious cases. Some critically ill patients and some mild cases may have been missed due to quarantine at other dedicated hospitals or community isolation centers.

## Conclusion

In conclusion, early comprehensive interventions including LPV/r-based treatment for COVID-19 pneumonia could be safely deployed. The median observed duration between symptom onset and viral PCR-negative conversion was 29 days. Patients with initial lower respiratory symptoms were associated with a prolonged positive PCR (> 28 days). Antiviral treatment on CT documented pneumonic patients may hasten negative conversion. A future randomized controlled trial of early administration of antiviral drugs is needed to validate these findings. Oxygen saturation should be monitored closely in older patients due to increased risk of disease progression.

## Data Availability

The datasets used and/or analysed during the current study are available from the corresponding author on reasonable request.

## Abbreviations

LPV/r: Lopinavir/ritonavir
CT: Computed tomography
OR: Odds ratio
CI: Confidence interval
PCR: Polymerase chain reaction
COVID-19: Coronavirus disease 2019
HLIU: High-level negative pressurized isolation unit
MEWS: Modified early warning score
ICU: Intensive care unit
ED: Emergency department
LDCT: Low-dose computed tomography
SARS-CoV-2: Severe acute respiratory syndrome coronavirus 2
HFNC: High-flow nasal cannula
GGO: Ground glass opacities
SD: Standard deviation
IQR: Inter-quartile ranges
